# The efficacy and safety of platelet-rich fibrin for rotator cuff tears: a meta-analysis

**DOI:** 10.1186/s13018-018-0881-3

**Published:** 2018-08-13

**Authors:** Xiu-hua Mao, Ye-jun Zhan

**Affiliations:** 1 Department of Pain Treatment, Ningbo No.2 Hospital, Ningbo, 315000 Zhejiang China; 2 Physical Health and Sports, College of Education, Lishui University, 1. No, Xueyuan Road, Liandu District, Lishui City, 323000 Zhejiang China

**Keywords:** Rotator cuff tears, Platelet-rich fibrin, Meta-analysis

## Abstract

**Background:**

The aim of this meta-analysis was to evaluate the efficacy and safety of platelet-rich fibrin (PRF) in improving clinical outcomes in rotator cuff tears.

**Methods:**

We searched the following databases: Pubmed, Embase, and Cochrane library databases from inception to April 2018. Studies that compared platelet-rich fibrin versus placebo for rotator cuff tears were included in this meta-analysis. Risk ratio (RR) with 95% confidence interval (CI) was pooled for discontinuous outcome, and weighted mean difference (WMD) with 95% CI was pooled for continuous outcome. Stata 12.0 was used for meta-analysis.

**Results:**

A total of eight studies with 219 patients were finally included in this meta-analysis. Compared with the control group, PRF has a negative role in reducing the re-tear rate (RR = 1.30, 95% CI = 0.97 to 1.75; *P* = 0.082). Subgroup analysis of re-tear rate was consistent in all subgroup analyses (single row or double row, volume, and risk of bias). There was no significant difference between the American Shoulder and Elbow Surgeons scale, University of California at Los Angeles scale, Constant score, and side effect (*P* > 0.05).

**Conclusion:**

In conclusion, our meta-analysis suggests that the PRF does not have better effect on improving the overall clinical outcomes and re-tear rate in the arthroscopic repair of rotator cuff tears.

## Introduction

Rotator cuff tears are one of the most common disorders of the shoulder with 250,000 to 300,000 rotator cuff repairs being performed annually in the USA [1]. Rotator cuff tears have a significant effect on daily life due to shoulder pain, range of motion decreased, and function loss [2]. Arthroscopic rotator cuff repair has become popular for orthopedic surgeons to improve patient outcomes and quality of life. However, a high re-tear rate was still a concern for extensively clinical use.

The reason for re-tear was that at the repair site, inferior fibrovascular tissue rather than native fibrocartilage tissue was formed, and thus, the repair site was exposing the insertion site to high stresses and increasing the risk of re-tear [3, 4]. In the past decades, some strategies, like the “transosseous-equivalent” suture-bridge technique, have been investigated for the treatment of rotator cuff tears to promote healing, but the outcomes were not promising enough.

Nowadays, the repair of rotator cuff tendon to bone is raising more and more interest. Lately, many growth factors were reported to be effective on the proliferation and collagen secretion of tenocytes in vitro. These growth factors could increase the biomechanical strength and promoted the tendon-to-bone healing in vivo. Many growth factors such as bone morphogenetic proteins (BMPs), basic fibroblast growth factor (bFGF), platelet-derived growth factor (PDGF), vascular endothelial growth factor (VEGF), insulin-like growth factor 1 (IGF-1), and transforming growth factor-b (TGF-b) have shown to be promising agents for rotator cuff tears in vivo and in vitro [5, 6].

Platelet-rich plasma (PRP) is a whole-blood fraction containing high platelet concentrations, which can release various growth factors mentioned above to promote healing [7]. Studies have reported that PRP can be used in the management of tendinopathy [8, 9]. But of legal restrictions on blood handling, a new family of platelet concentrate appears in France, which is called platelet-rich fibrin (PRF) [10]. PRF, unlike other platelet concentrates, can progressively release cytokines during fibrin matrix remodeling.

Therefore, applying growth factor mixtures through platelet-rich fibrin maps a promising future for tendon-bone insertion regeneration like rotator cuff repair. In fact, these technologies have been applied on treating chronic tendinopathy [11], bone healing [12], acute ligament repair [13], and tendon repair [14].

Additionally, these products were approved by the US Food and Drug Administration (FDA) for clinical use. Although approved, these products have not been required by the FDA to show efficacy. Nevertheless, to our best knowledge, none of the previous studies, which involved a large number of patients, has been performed to investigate the efficacy of rotator cuff repair with or without PRF by analyzing clinical and radiological outcomes.

The aim of the present meta-analysis was to assess whether administration with PRF has a beneficial role in improving clinical outcomes and side effect during the arthroscopic repair of rotator cuff tears.

## Methods

### Search strategies

Two reviewers searched the Pubmed, Embase, Web of Science, and Cochrane library independently (Xiu-hua Mao and Ye-jun Zhan) from inception to April 2018. The following keywords and Mesh terms were used for searching: “rotator cuff,” “rotator cuff tears,” “Rotator Cuff Injuries”[Mesh] “platelet rich fibrin,” “platelet rich,” “PRF,” “platelet rich fibril matrix,” “PRFM,” and “Platelet-Rich Fibrin”[Mesh]. Publication language was restricted to English. Reference list in systematic review or meta-analysis was also manually searched to avoid omitting any relevant studies.

### Inclusion criteria

The inclusion criteria were as follows:

Participant (P): arthroscopic rotator cuff repair as regards the age and sex.

Intervention (I): administration with PRF as the intervention group.

Comparison (C): placebo or saline as the control group.

Outcomes (O): re-tear rate, American Shoulder and Elbow Surgeons scale (ASES), University of California at Los Angeles scale (UCLA), Constant score, and adverse effect.

Study (S): only RCTs were included in this meta-analysis.

### Data extraction and quality assessment

Two readers (Xiu-hua Mao and Ye-jun Zhan) independently extracted all the data as follows: general characteristics (no. of patients, mean age, country, intervention, follow-up, and outcomes). The methodological quality of the trials was assessed using the *Cochrane Handbook for Systematic Reviews of Interventions 5.3*. A total of seven items were included to assess the quality of study: random sequence generation, allocation concealment, blinding to the participant, blinding to outcome assessment, incomplete outcome, selective reporting, and other potential bias. Each item was assessed as “low,” “unclear,” and “high.”

### Statistical analysis

We used Review Stata 12.0 to perform statistical analysis. For continuous variables, we used the weighted mean difference, whereas for those categorical dichotomous, we used relative risks (RR) to analyze, and 95% confidence intervals (CI) were reported in analysis of both continuous and dichotomous variables. *P* value beneath 0.05 was considered to be statistically significant. Homogeneity was tested by the *Q* statistic (significance level at *P* = 0.10) and the *I*^2^ statistic (significance level at *I*^2^ = 50%). A random-effects model was used if the *Q* or *I*^2^ statistic was significant; otherwise, a fixed-effects model was used. Subgroup analysis were performed in the analysis of re-tear rate according to the operative technique (single row or double row), risk of bias (low or unclear/high), volume of PRP (< 5 or ≥ 5 ml), follow-up duration (< 15 or ≥ 15 months), and size of rotator cuff tears (small-medium or large-massive). Sensitivity analysis was performed based on omitting one study in turn to investigate the influence of a single study on the overall RR estimates. Publication bias was not performed because the included studies were less than ten.

## Results

### Search results

**Fig. 1:**
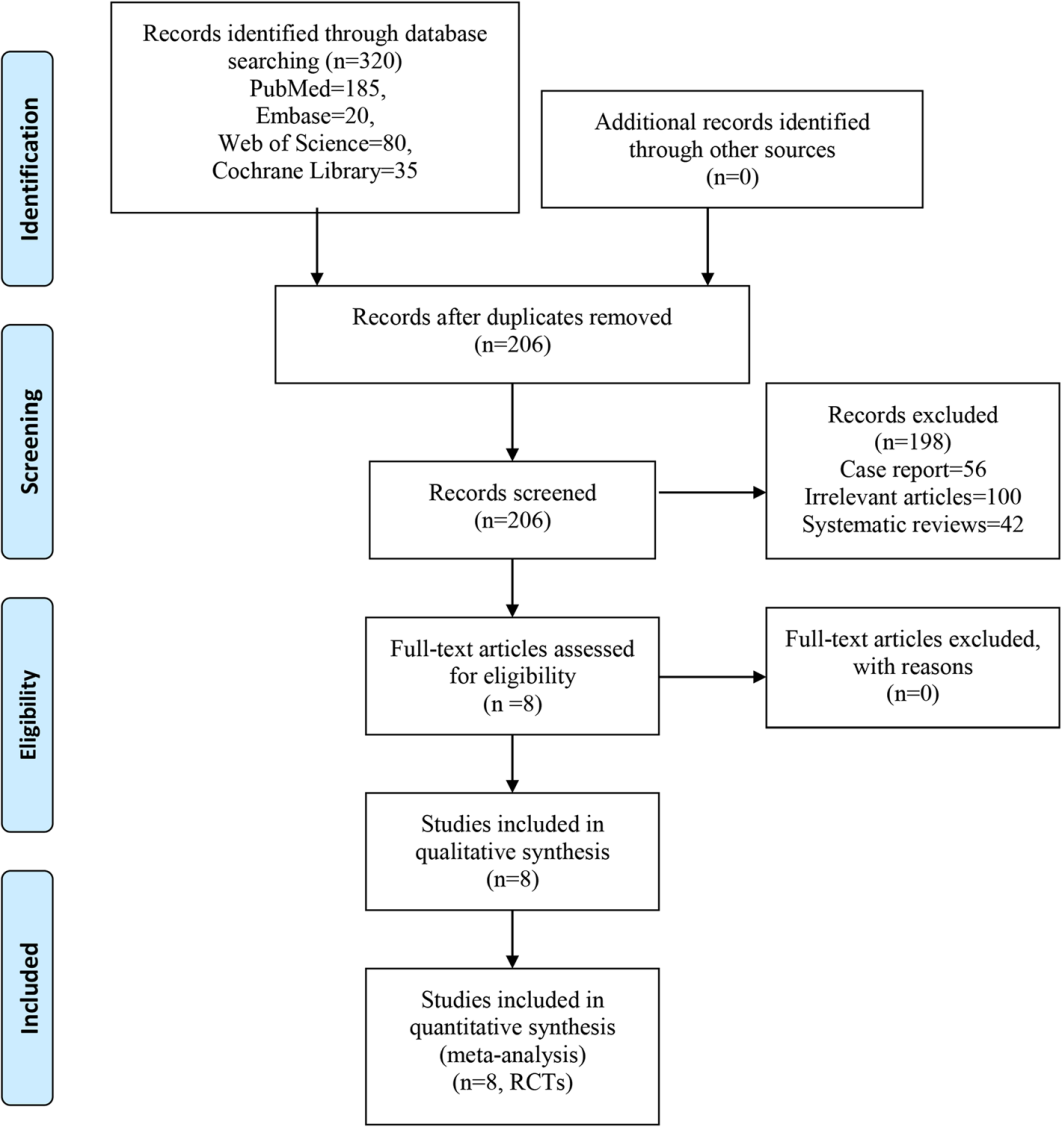
Flow diagram of the study selection process

Details of study identification, screening, and selection are given in Fig. 1. Firstly, we retrieved 320 relevant reports from electronic databases. And 114 papers were removed by Endnote software for duplications. Thus, 206 papers were screened for the next step. Then, according to the inclusion criteria, 198 records were excluded. Finally, eight RCTs [15–22] involving 364 patients (PRF = 177, control = 187) finally met the predetermined inclusion criteria and were included for final analysis.

### Demographic characteristics

We summarized the general characteristics of all the included studies and listed in Table 1. All of the included studies were published from the year 2011. Three studies were originated from the USA, two from Spain, two from Italy, and one from France. Only one study did not report the tear size, and the rest of the studies all reported the tear size. Mean age of the included patients ranged from 55.2 to 66. Sample size ranged from 14 to 43 with a total of 364 patients. Follow-up duration ranged from 12 to 27.2 months.

**Table 1:** General characteristics of the included studies. 1, re-tear rate; 2, ASES; 3, UCLA; 4, Constant score; 5, adverse event

Study	Country	Participants	Surgical procedure	Mean age		No. of patients		Follow-up (months)	Outcomes
				PRF	Control	PRF	Control		
Antuna 2013	Spain	Massive full-thickness rotator cuff tears	Double-row techniques	NS	NS	14	14	24	1, 4
Bergeson 2012	USA	Full-thickness rotator cuff tears	Single- or double-row techniques	65	65	16	21	27	1, 2, 3, 5
Rodeo 2012	USA	Full-thickness rotator cuff tears	Single or double-row techniques	58.9	57.2	19	22	19	1, 2, 5
Weber 2013	USA	Full-thickness rotator cuff tears	Single-row techniques	59.7	64.5	29	30	12	1, 2, 3, 4, 5
Castricini 2011	Italy	NS	Double-row technique	55.5	55.2	43	45	20.2	1, 5
Gumina 2012	Italy	Large full-thickness posterosuperior rotator cuff tear	Single-row technique	60	63	39	37	13	4, 5
Márquez 2011	Spain	Massive rotator cuff tear of at least 5 cm and including 2 tendons	Single-row technique	65	NS	14	14	12	1, 2, 4
Zumstein 2016	France	Full-thickness rotator cuff tears	Single- or double-row techniques	65	66	17	18	12	1, 5

**Fig. 2:**
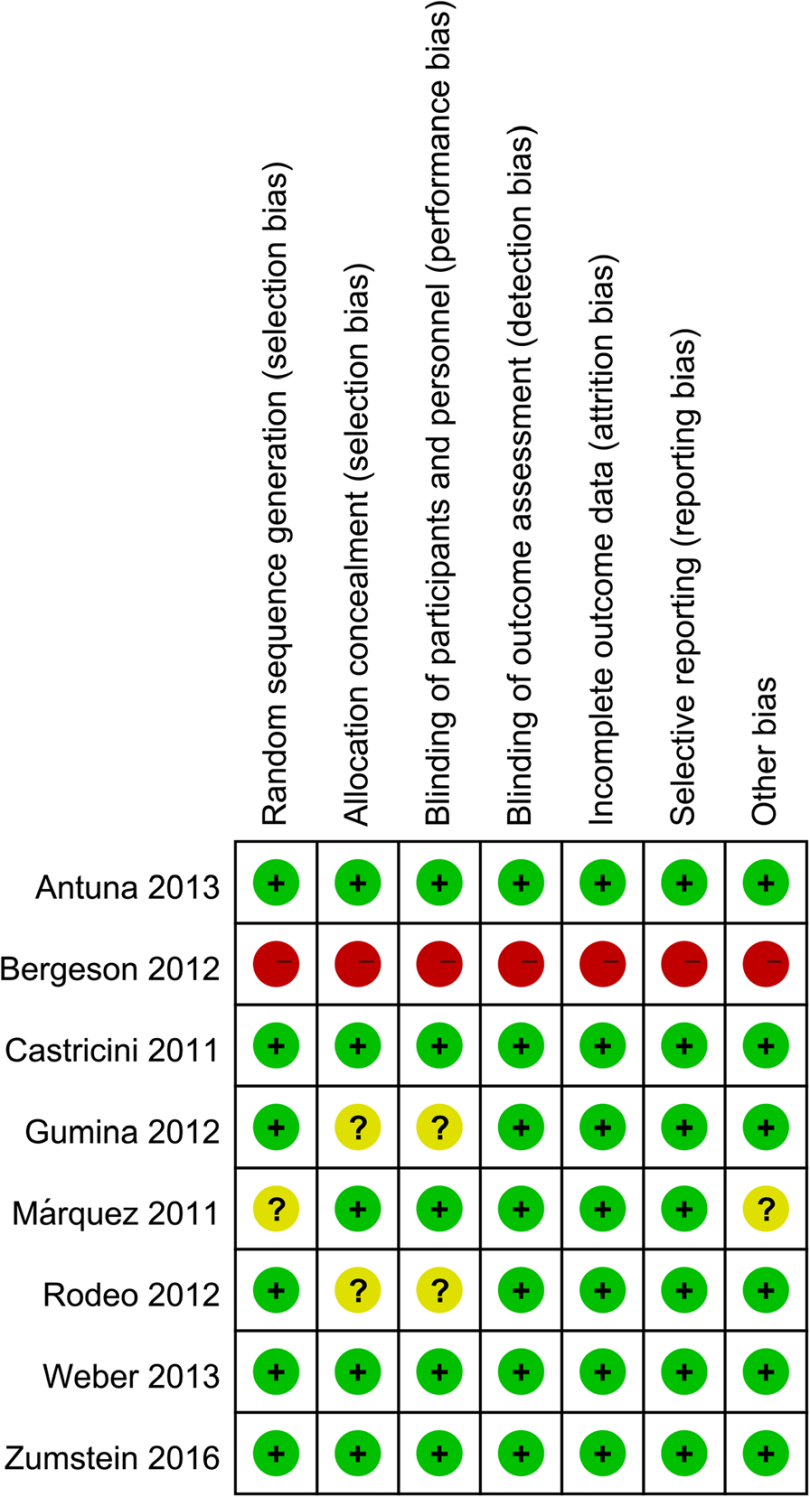
Risk of bias summary

**Fig. 3:**
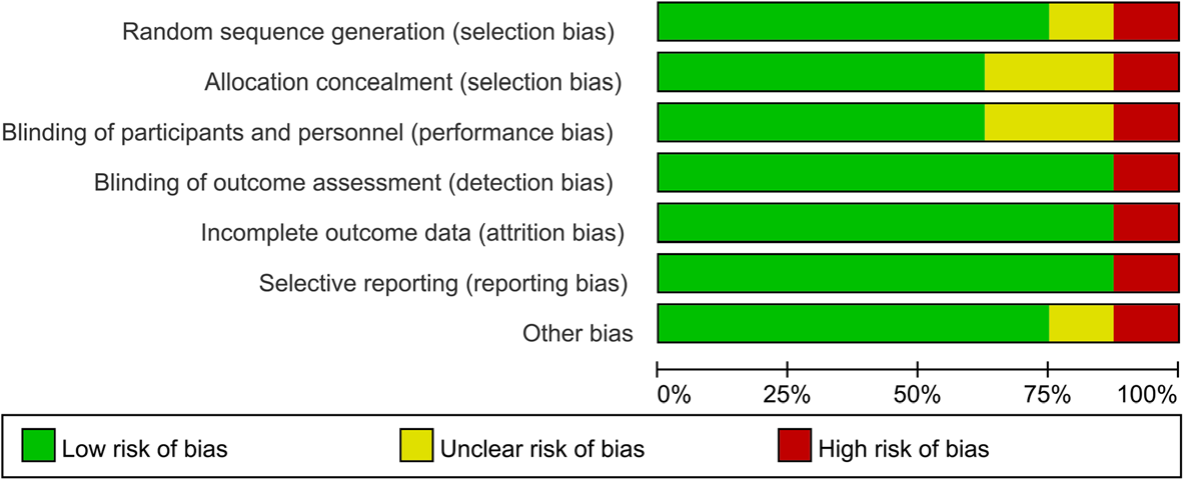
Risk of bias graph

Figures 2 and 3 present the risk of bias summary and risk of graph respectively. Six studies reported the random sequence generation and one with high risk of bias. Five studies were with low risk of bias, and two were with unclear risk of bias.

### Meta-analysis results

#### Re-tear rate

**Fig. 4:**
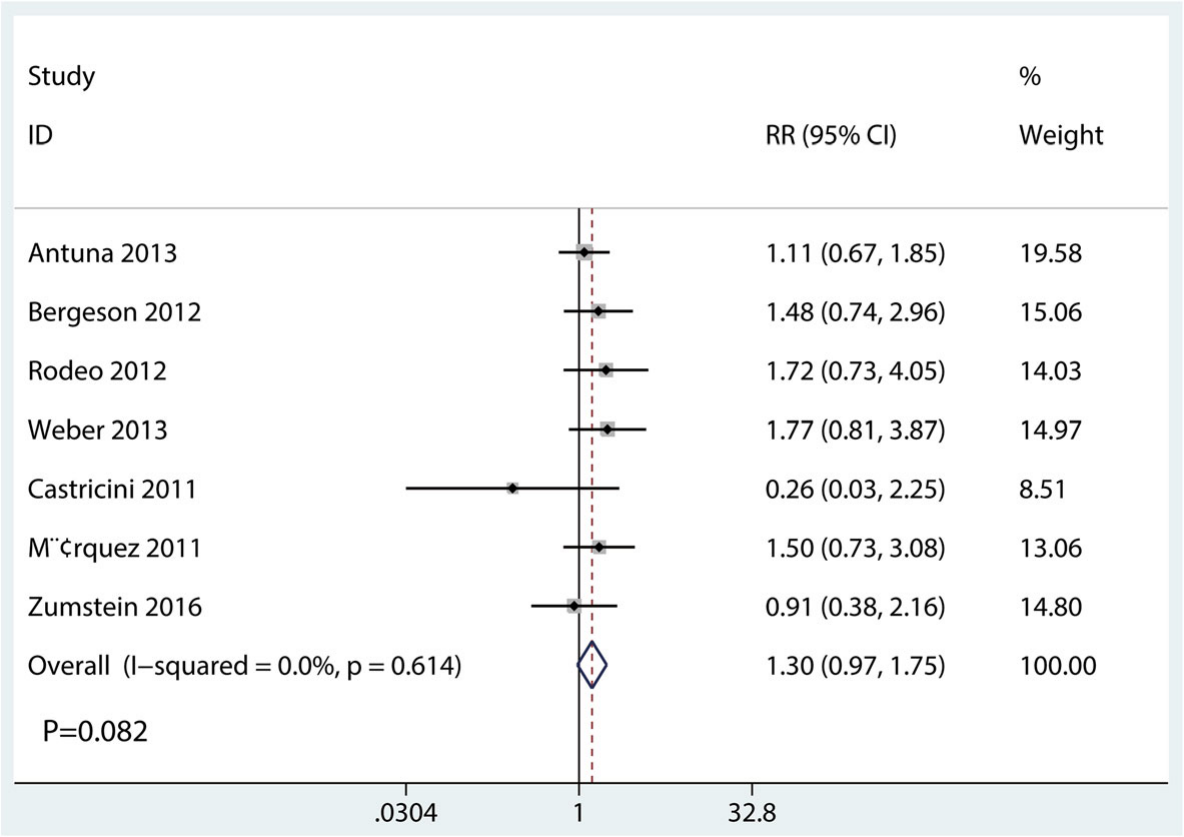
Forest plot for the comparison of re-tear rate between the PRF group and the control group

Seven studies [15–19, 21, 22] perform available data for postoperative re-tear rate. There was no heterogeneity across the included studies (*I*^2^ = 0.0%, *P* = 0.614). Compared with the control group, PRF group was not associated with a reduction of the re-tear rate (RR = 1.30, 95% CI = 0.97 to 1.75; *P* = 0.082, Fig. 4). Table 2 presents the results of subgroup analyses. The findings of re-tear rate were consistent in all subgroup analyses.

**Table 2:** Subgroup analysis for the re-tear rate

Subgroup	No. trials	Relative risk (95% CI)	*P* value	*I*^2^ (%)	Test of interaction, *P*
Total	7	1.30 (0.97, 1.75)	0.082	0.0	
Operative technique					
Single row	2	1.65 (0.82, 2.77)	0.069	0.0	0.106
Double row	2	0.87 (0.55, 1.39)	0.566	3.3	
Single or double row	3	1.60 (0.92, 2.77)	0.097	0.0	
Risk of bias					
Low	3	1.49 (0.99, 2.25)	0.058	0.0	0.098
Unclear/high	4	1.12 (0.73, 1.71)	0.607	7.1	
Volume					
< 5 ml	1	1.77 (0.81, 3.87)	0.150	–	0.152
≥ 5 ml	3	1.40 (0.94, 2.10)	0.097	0.0	
Unclear	3	0.99 (0.59, 1.67)	0.963	28.2	
Follow-up					
< 15 months	4	1.95 (0.87, 4.37)	0.103	0.0	0.105
≥ 15 months	3	1.37 (0.60, 3.10)	0.449	0.0	
Size of rotator cuff tears					
Small-medium	3	0.77 (0.31, 1.86)	0.271	0.0	0.226
Large-massive	4	1.72 (0.64, 4.28)	0.582	0.0	

#### ASES

**Fig. 5:**
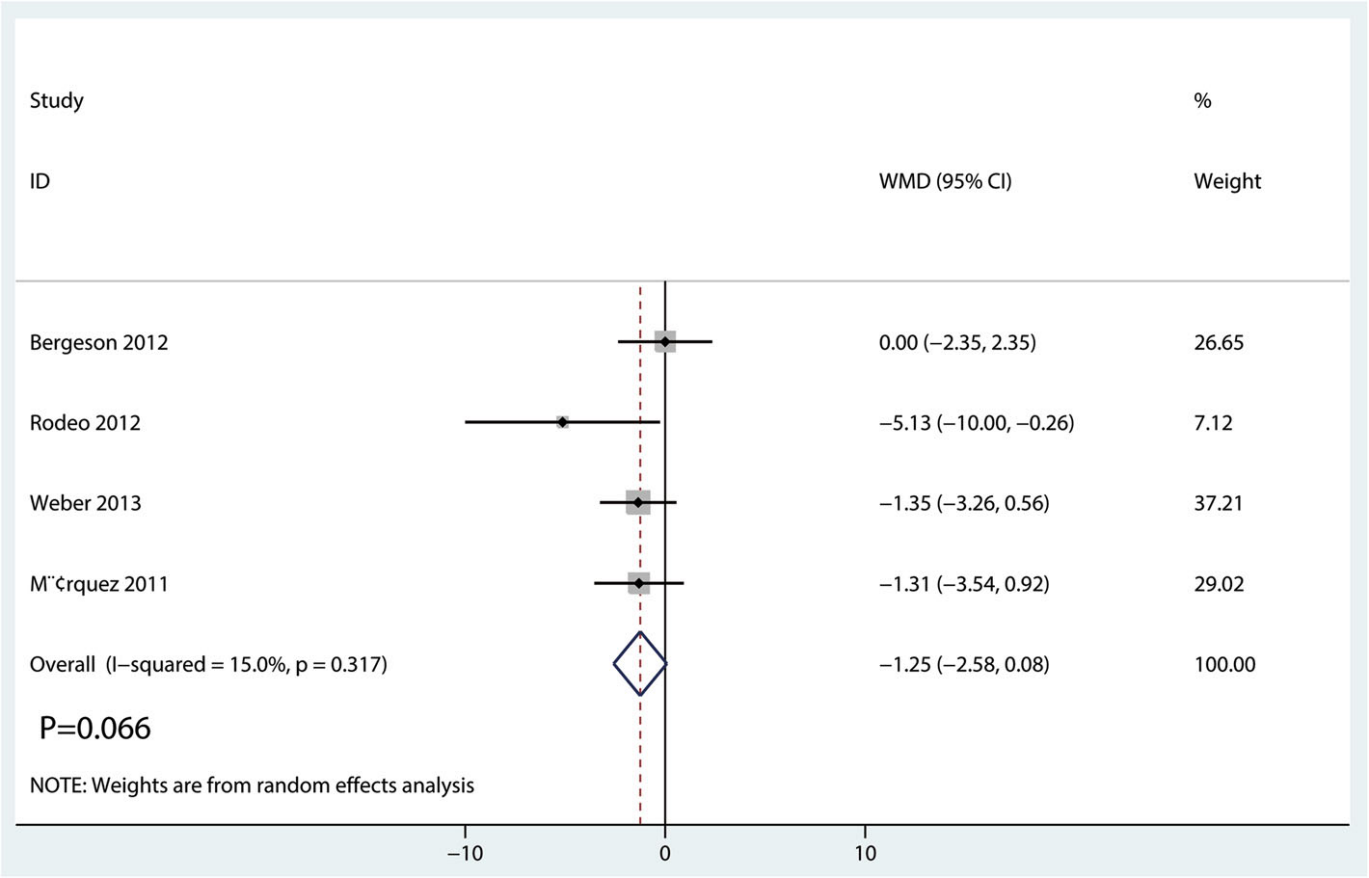
Forest plot for the comparison of ASES between the PRF group and the control group

Four studies [16–18, 21] reported postoperative ASES scores. There was little heterogeneity across the included studies (*I*^2^ = 15.0%, *P* = 0.317). There was no significant difference in ASES score between the PRF group and the control group (weighted mean difference (WMD) = − 1.25, 95% CI = − 2.58 to 0.08; *P* = 0.066, Fig. 5).

#### UCLA

**Fig. 6:**
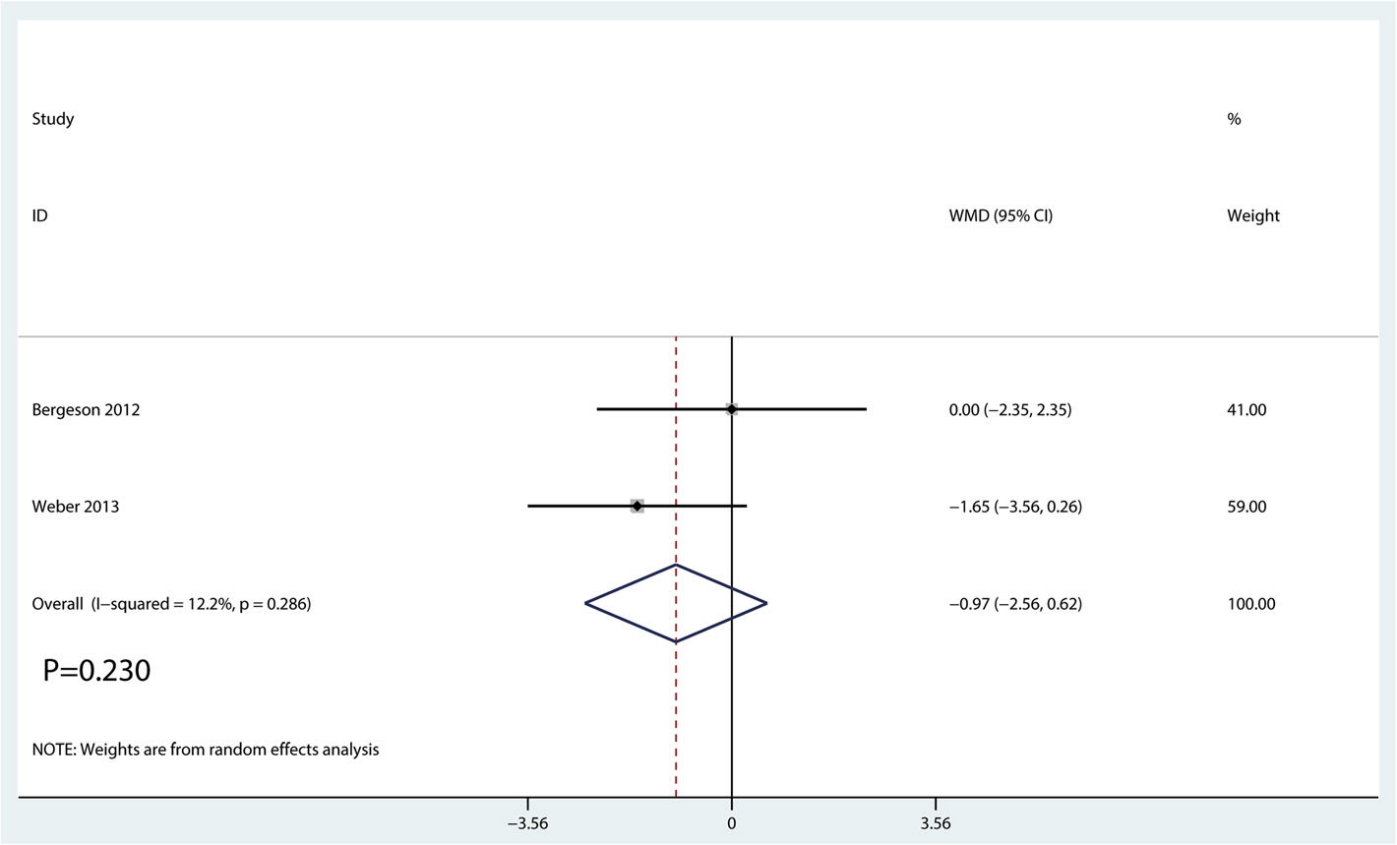
Forest plot for the comparison of UCLA between the PRF group and the control group

Two studies [16, 18] reported postoperative UCLA scores. There was no significant difference in UCLA score between the PRF group and the control group. The MD was − 0.96 (WMD = − 0.97, 95% CI = − 2.56 to 0.62; *P* = 0.230, Fig. 6).

#### Constant score

**Fig. 7:**
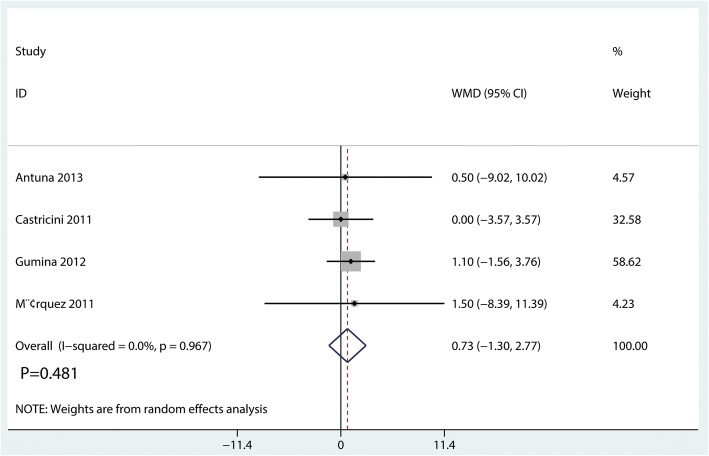
Forest plot for the comparison of Constant score between the PRF group and the control group

Four studies [15, 19–21] perform available data for postoperative constant score. There was no heterogeneity across the included studies (*I*^2^ = 0.0%, *P* = 0.967). Compared with the control group, PRF group was not associated with a reduction of the constant score (WMD = 0.73, 95% CI = − 1.30 to 2.77; *P* = 0.481, Fig. 7).

#### Side effect

**Fig. 8:**
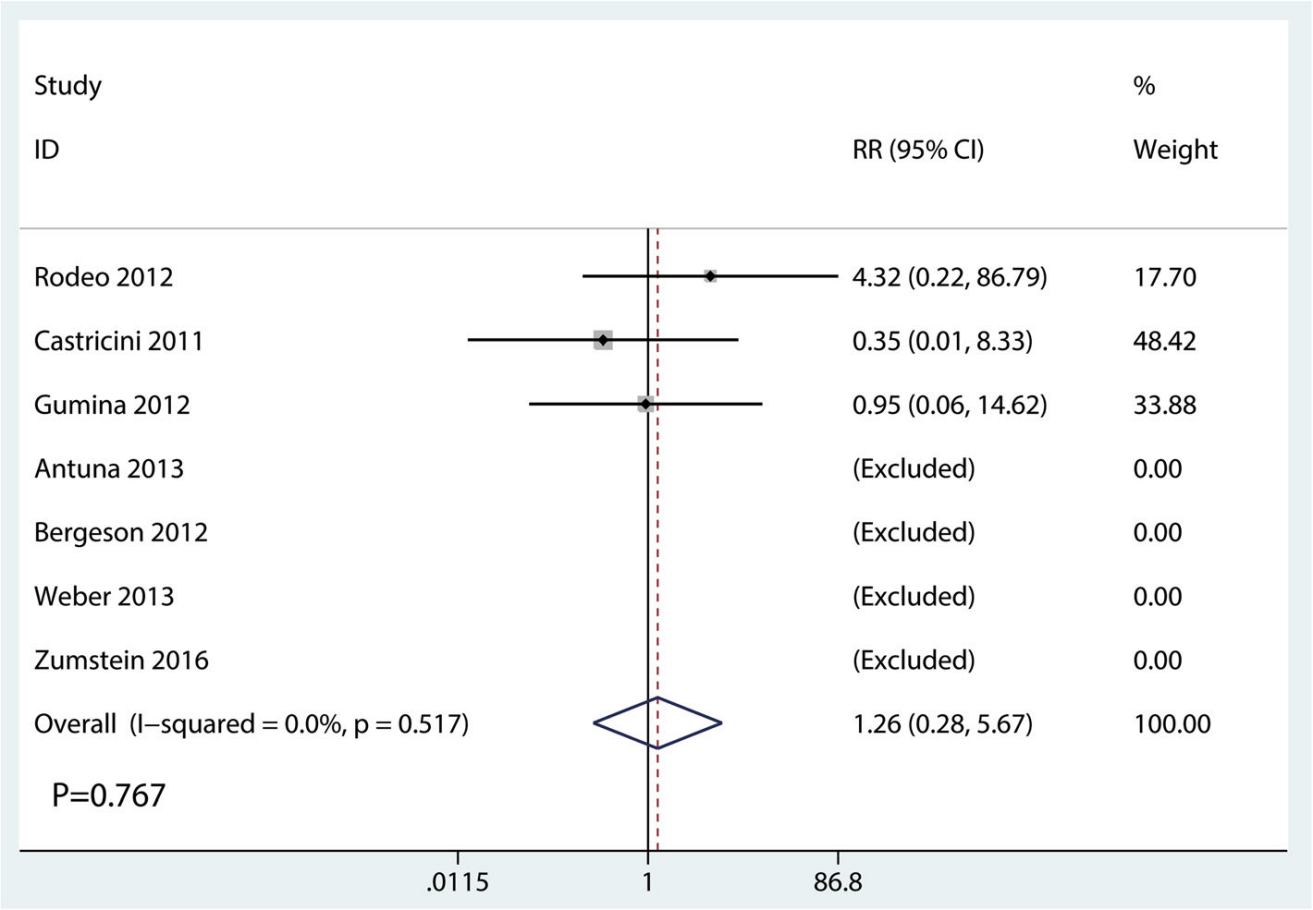
Forest plot for the comparison of side effects between the PRF group and the control group

A total of seven studies [15–20, 22] reported postoperative complication. The pooled result showed that there was no significant difference in the side effect between the PRF group and the control group (RR = 1.26; 95% CI = 0.28, 5.67; *P* = 0.767; Fig. 8).

## Discussion

### Main findings

Our meta-analysis comprehensively and systematically reviewed the current available literature and found that (1) PRF compared with placebo did not significantly reduced re-tear rate for rotator cuff tear patients; the evidence of the re-tear rate was consistent in most subgroup analyses and was confirmed by TSA; (2) PRF has no benefit on the shoulder function at the final follow-up when compared with placebo; (3) PRF was not associated with an increase of the complications than the control group.

### Comparison with other meta-analyses

Only one relevant meta-analysis on the topic has been published [23]. Several differences between ours and the previous ones should be noted. First, the previous ones mixed PRP and PRF in the same intervention group and thus cause large heterogeneity across the studies. Second, two studies were not included in the previous meta-analysis and the publication bias was inevitable. Andia et al. [24] conducted a review about the PRP therapy for tendinopathy, plantar fasciopathy, and muscle injuries. Results showed that PRP therapies were useless. Meanwhile, Andia et al. [25] revealed that PRP has no effects on muscle injury and tendinopathy.

The current meta-analysis systematically scanned all of the available studies and has given a relative credible evidence for the clinical effects of PRF on rotator cuff tear patients. In this meta-analysis, we identified re-tear rate as the primary outcome. Results showed that PRF has a negative effect on the overall incidence of re-tear at the final follow-up. Previous meta-analysis did not pool this important outcome [23]. Re-tear could make the patients dissatisfied and increase additional costs. Subgroup analysis indicated that PRF has a positive role in reducing the incidence of re-tear rate than the control group. However, long-term effects of PRF were extremely important for clinical administration.

Hueley et al. [23] conducted a meta-analysis, and the pooled result was similar with our meta-analysis. PRF is considered as one kind of platelet concentrates, and its molecular structure with low thrombin concentration is an optimal matrix for migration of endothelial cells and fibroblasts, which can progressively release several cytokines to help fibrin matrix remodeling.

In an animal experiment, we found that PRF has a beneficial role in tissue regeneration whereas there was a negative role in a clinical experiment [19]. Randelli et al. [26] reported that autologous PRP reduced pain in the first postoperative months and affected cuff rotator healing for both grade 1 and 2 tears. Furthermore, Andia et al. [27] revealed that PRP, as an autologous biotechnology product, has a positive effect on experimental tendon healing.

The reason for the failure of PRF to fulfill its promise remains unclear. There are some possible interpretations for this phenomenon. On the one hand, patients all received autologous source PRF and the growth factors contained in PRF vary from person to person, for which there were much more difficulty for experimentally bias control. To be specific, there is a chance that the patient’s blood plasma contains excessive TGF-β, and its potential effect on exuberant fibrosis may affect the therapeutic effect of PRF. Nevertheless, some patients’ plasma may contain abundant inflammatory mediators, which could adversely affect healing process. More importantly, none of us has enough data to determine the best clinical usage of PRF products. And there were some prior articles that noticed this problem [27]. On the other hand, platelet-rich products may also influence the effect.

For example, recent studies showed that not all separation systems yield a similar product, because there are many factors that can influence the separation, including the volume of blood, single- versus double-spin cycles, centrifuge rates, the need for an activator, white blood cell concentrations, and the final platelet and growth factor concentrations. In other words, different products can have varied platelet concentrations, and therefore, platelet-derived growth factor concentrations may differ between various systems [28]. Additionally, it is also possible that the clot may occupy the space between the tendon and bone, resulting in a gap. Once the material dissolves, they may inhibit the healing process.

Moreover, although patients all received autologous source PRF, these procedures are not absolutely safe. Some postoperative complications seem to be related with PRF. The most common one is infection. Even though it is performed with aseptic techniques, the PRF group has a higher infection rate than the control group [29]. The cause of infection is unclear, but multiple steps obliged to prepare PRFM require additional interactions between sterile and non-sterile fields and introduce variables, increasing infection risk. However, we did not find a significant difference in postoperative complication between the two groups in our meta-analysis.

Several limitations also existed in this meta-analysis: (1) initial tear size was not compared between the PRF and control group; (2) PRF volume, platelet concentration, and activating agent were different in the included studies, and thus, clinical heterogeneity was large in the outcomes; (3) the follow-up period varied among included studies, and thus, clinical effects of PRF in the same follow-up period need to be further confirmed; (4) sample size was relatively small in the included studies, and thus, high quality with large-scale sample RCTs were needed.

## Conclusion

In conclusion, this meta-analysis suggests that the PRF has no benefits on the overall clinical outcomes and re-tear rate for the arthroscopic repair of full-thickness rotator cuff tears. But, given all the shortness that this meta-analysis has, further research and analysis are required to make a more reliable conclusion.

## Abbreviations

ASES: American Shoulder and Elbow Surgeons scale
bFGF: Basic fibroblast growth factor
BMPs: Bone morphogenetic proteins
IGF-1: Insulin-like growth factor 1
PDGF: Platelet-derived growth factor
PRF: Platelet-rich fibrin
PRP: Platelet-rich plasma
TGF-b: Transforming growth factor-b
UCLA: University of California at Los Angeles scale
VEGF: Vascular endothelial growth factor
